# Predictors of contraceptive discontinuation among postpartum women in Arusha region, Tanzania

**DOI:** 10.1186/s40834-021-00157-6

**Published:** 2021-05-03

**Authors:** Michael J. Mahande, Ryoko Sato, Caroline Amour, Rachel Manongi, Amina Farah, Sia E. Msuya, Bilikisu Elewonibi, Iqbal Shah

**Affiliations:** 1 Department of Epidemiology and Biostatistics, Institute of Public Health, Kilimanjaro Christian Medical University College, P.O Box 2240, Moshi, Tanzania; 2 Department of Global Health and Population, Harvard T.H. Chan School of Public Health, Boston, USA; 3 Department of Community Health, Institute of Public Health, Kilimanjaro Christian Medical University College, P.O Box 2240, Moshi, Tanzania; 4 Department of Community Health, KCMC Hospital, P.O Box 3010, Moshi, Tanzania

**Keywords:** Contraceptive, Discontinuation, Determinants, Tanzania

## Abstract

**Background:**

Postpartum contraceptive discontinuation refers to cessation of use following initiation after delivery within 1 year postpartum. Discontinuation of use has been associated with an increased unmet need for family planning that leads to high numbers of unwanted pregnancies, unsafe abortion or mistimed births. There is scant information about contraceptive discontinuation and its predictors among postpartum women in Tanzania. This study aimed to determine predictors of contraception discontinuation at 3, 6, 12 months postpartum among women of reproductive age in Arusha city and Meru district, Tanzania.

**Methods:**

This was an analytical cross-sectional study which was conducted in two district of Arusha region (Arusha city and Meru district respectively). A multistage sampling technique was used to select 13 streets of the 3 wards in Arusha City and 2 wards in Meru District. A total of 474 women of reproductive age (WRAs) aged 16–44 years residing in the study areas were included in this analysis. Data analysis was performed using STATA version 15. Odds ratios (ORs) with 95% confidence interval (CI) for the factors associated with contraceptives discontinuation (at 3, 6 and 12 moths) were estimated in a multivariable logistic regression model.

**Results:**

Overall, discontinuation rate for all methods at 3, 6, and 12 months postpartum was 11, 19 and 29% respectively. It was higher at 12 months for Lactational amenorrhea, male condoms and injectables (76, 50.5 and 36%, respectively). Women aged 40–44 years had lower odds of contraceptive discontinuation at 3 months as compare to those aged 16 to 19 years. Implants and pills users had also lower odds of contraceptive discontinuation compared to injectable users at 3, 6 and 12 months respectively.

**Conclusion:**

Lactational amenorrhea, male condoms and injectables users had the highest rates of discontinuation. Women’s age and type of method discontinued were independently associated with postpartum contraceptive discontinuation. Addressing barriers to continue contraceptive use amongst younger women and knowledge on method attributes, including possible side-effects and how to manage complications is warranted.

## Introduction

Contraceptive discontinuation is defined as starting contraceptive use and then stopping for any reason while still at risk of an unintended pregnancy [1]. It has been reported to be higher for short-acting methods such as condoms, injectables, pills and traditional methods as they can be discontinued by the user herself compared to long- acting reversible methods such as implants and the Intrauterine device (IUD) which require a visit to facility to discontinue [1–3]. The method-related reasons and contraceptive failure have been reported as the predominant causes for contraceptive discontinuation [4].

Postpartum period in the first 12 months following childbirth has been associated with high unmet need for contraceptives coupled with unintended pregnancies [5]. Breastfeeding practices and beliefs about return of menses as a marker of fertility resumption during the postpartum period, makes it difficult for women to determine their fertility risk. Thus, women are less motivated to start contraceptive use while breastfeeding [6, 7]. Previous investigators have demonstrated that women who discontinue contraception use during the postpartum period may opt not to use any method (contraceptive discontinuation) or switch to different modern method (method switching) which is less effective than the previous method at preventing pregnancy, and thereby exposes women to risk of unintended pregnancy, abortions and mistimed pregnancies/births [7, 8].

Sustaining postpartum contraception use is important for woman’s fertility because it ensures optimal birth spacing, prevents unintended pregnancies, abortion and has an impact on infant and child survival [9]. Examining postpartum contraceptive discontinuation will shed a light on the knowledge gaps in contraceptive use such as trends and determinants for contraceptive discontinuation, and will help in reducing unmet need for family planning [10]. It will also provide evidence for areas that require coordinated efforts between different stakeholders involved in family planning programs and the government. This will help to improvement quality of services for family planning and reduces the discontinuation postpartum contraception.

Previous studies from developing countries showed that, on average, 19–64% of women discontinued using reversible contraceptive methods by the 12th month of use [4, 11]; [12–15]. The discontinuation rate for condoms within the first 12 months is higher than intrauterine devices (50% vs 13%, respectively), and up to 40% higher for other methods such as pill, injectable, periodic abstinence and withdrawal [3, 4, 16]. Contraceptive discontinuation among women with no desire to get pregnant increases the risk for unwanted pregnancies [8]. This reflects a failure of family planning programs and services [3, 4, 17–19].

According to Tanzania Demographic and Health Survey, only 15.5 and 22.4% of women reported using modern contraceptive methods at 3 months and 12 months post-delivery respectively, especially during postnatal visit probably due to contraceptive counselling [20]. A cohort study conducted in Northern Tanzania among 5284 pregnant women who were followed from 6 to 15 months postpartum, reported that 34% of women initiated contraceptive use during the postpartum period and 25% of the participants started at 7 months postpartum [21]. Authors in this study noted that 18.8% of contraceptive users discontinued at 15 months postpartum. The reasons for contraceptive discontinuation include partner disapproval (32%), side-effects (6%), wanting a child (4%) and other reasons (37%) [21]. The most recent study in Tanzania reported that short contraceptive methods were associated with high rate of discontinuation compared to long term acting contraceptives [13].

There is scant information on contraceptive discontinuation rates, patterns and associated factors post-delivery. This study aimed to determine predictors of contraception discontinuation at 3, 6, 12 months postpartum among women of reproductive age in Arusha city and Meru district, Tanzania.

## Methods

### Study design and setting

This study was conducted in two districts of Arusha region (Arusha city and Meru district respectively) from December 2017–June 2018.

### Study population, sample size and sampling method

**Fig. 1:**
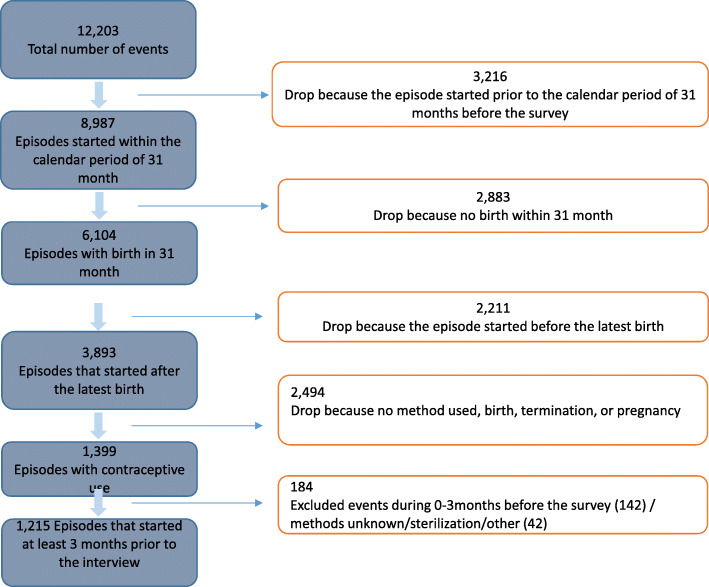
Flowchart of Events included in Contraceptive Calendar

The study sample included women of reproductive age (WRAs) aged 16–44 years who started to use family planning methods (modern/traditional) after a delivery (6 weeks) that occurred at least 12 months prior to the survey. Multistage cluster sampling with probability proportional to size was used to draw respondents from 13 streets (i.e. 3 wards) in Arusha City and 2 wards in Meru District. The details of the sampling procedures have been described elsewhere [15, 22]. A contraceptive calendar is a contraceptive history collected for each woman who, or whose husband, was not sterilized at the calendar’s start. The data were recorded in a calendar matrix, consisting of rows and columns, with each row of the calendar representing a particular month. The sample size was estimated based on number of events included in the contraceptive calendar, which included retrospectively reported month-by-month during 31 months before the interview. A total of 12,203 contraceptive use events were recorded during the study period. We excluded 3216 episodes which started prior to the calendar period of 31 months before the survey; 2883 episode because no birth within 31 month; 2211 episode because the episode started before the latest birth; 2494 episode because no method used, birth, termination, or pregnancy; 184 events during 0-3 months before the survey [methods unknown (*n* = 142) & sterilization/other (*n* = 42)]. The remaining 1215 episodes of use within the last 3 to 28 months prior to interview constituted the final sample size (Fig. 1).

### Study variables

The main outcome was contraceptive discontinuation after starting to use of contraceptive at three time points; 3 months, 6 months, and 12 months post-delivery. The independent variables were selected based on the previous studies. These include sociodemographic characteristics: maternal age, maternal education and wealth index. Women were asked of the number of antenatal care (ANC) visits during the last pregnancy, number of months since birth to first family planning use, received contraceptive counseling during ANC or PNC visits and attendance of postnatal care after last delivery. The type of contraceptive methods used were IUD, pills, male condoms, injectables, implant, rhythm and withdrawal. Women were also asked for the reasons related to discontinuation of modern contraceptive.

### Data collection methods

Data collection was conducted through face-to-face interviews using questionnaire administered using tablets. A team of trained research assistants including medical doctors, statisticians, demographers and social scientist were used to collect information from the participants. A standardized questionnaire in Kiswahili language which was adapted from the Tanzania Demographic and Health Survey (TDHS) 2015–2016 [12] was used to collect information from the study participants for household survey to meet the WIE objectives. These variables includes women age, education level, marital status, area of residence, wealth index, utilisation of contraceptive methods, cost to access the family planning services, type of facility, distance to health facility, type of contraceptive used and availability of family planning commodities to mention a few.

### Data analysis

Data analysis was performed using STATA version 15. Descriptive statistics were summarized using frequency and proportions for categorical variables. The percentage of women who had discontinued their method of contraception at 3, 6 and 12 months were reported. Adjusted Odds ratios (ORs) with 95% confidence interval (CI) for the factors associated with contraceptive discontinuation were estimated in a multivariable logistic regression model. The correlation between discontinuation at certain time (3, 6, and 12 months) with sociodemographic characteristics were separately evaluated. We used the logistic regression because the dependent variable is a dummy variable (discontinued at 3, 6, or 12 months). Independent variables we used for the regression analysis were reported in Table 1. Only variables which were significant in the bivariate analysis were included in the final model.

**Table 1:** Characteristics of study participants (*N* = 1215 episodes)

Characteristics	n	%
FP counselling at ANC (Yes)		
Yes	382	70.9
No	157	29.1
Family Planning counselling at postnatal care		
Yes	424	76.5
No	130	23.5
Mother age (year)		
16–19	10	1.7
20–24	161	28.0
25–29	181	31.5
30–34	111	19.3
35–39	80	13.9
40–44	31	5.4
Education level		
None	17	3.0
Primary	316	55.1
Secondary	209	36.4
Higher education	32	5.6
Wealth index (as per DHS)		
Poorest	112	19.5
Poorer	59	10.3
Medium	101	17.6
Richer	253	44.1
Richest	49	8.5
Method discontinued		
Injectables	175	30.5
Implants	153	26.7
Pills	61	10.6
Condom	22	3.8
Rhythm	79	13.8
Other	84	14.6
Sample = 574 women with some FP use that initiated at least 12 months prior to the survey		
Number of living children		
0	2	0.3
1	201	35.0
2	165	28.7
3	117	20.4
4	60	10.5
5	20	3.5
6	6	1.0
7	2	0.3
Desire to get pregnant		
No	176	30.6
Yes	398	69.34
Received FP counselling during their ANC or PNC		
No	460	80.1
Yes	114	19.9
Support from the husband/partner for contraceptive use		
No	125	21.8
Yes	449	78.2

## Results

### Characteristics of study participants

We have a total of 1215 episodes from the calendar data (Table 1). About one third of all episodes (31%) were aged 25 to 29 years old. More than half (55%) of all episodes had primary education. Majority (71%) of all episodes were reported to have received family planning counselling during ANC visit and postnatal care visit (76.5%).

### Contraceptive discontinuation at 3, 6 and 12 months postpartum

Overall, 11.5% of all episodes discontinued at 3 months. Of these, injectables, pills and male condoms were discontinued by 16, 16 and 9% of the sample respectively, while implants and IUD discontinued by 1.55 and 3.23% at 3 months. On the other hand, of the 11.5% episodes discontinued at 3 months, 27% were LAM users (Table 2). Likewise, there was 19.4% episodes discontinued at 6 months, where injectables, pills and male condoms were discontinued by 24.64, 25.44 and 20.37% of the sample respectively while implants and IUD accounted for 3.84 and 8.34% during the same period. At 6 months, 49% of those 19.4% episodes discontinued were LAM users (Table 3). Furthermore, about 29.4% episodes discontinued at 12 months, injectables, pills and male condoms accounted for 38.15, 36.48% and 50. 49% of the sample while implants and IUD contributed 5.11 and 9.06% at 12 months while 76.12% of 29.4% episodes discontinued at 12 months were LAM (Table 4).

**Table 2:** Contraceptive discontinuation rates and associated reasons (*N*=1,215 episodes)

At 3 months postpartum									
Reasons for discontinuation									
Contraceptive methods	Method failure	Desire to become pregnant	Other fertility related reasons	Side effects	Wanted more effective method	Other method related	Other/DK	All reasons	Unweighted N
IUD	0.00	0.00	0.00	3.23	0.00	0.00	0.00	3.23	73
Injectables	0.29	0.27	1.96	10.89	0.80	1.12	1.12	16.45	351
Implants	0.00	0.00	0.00	1.55	0.00	0.00	0.00	1.55	359
Pill	0.00	0.00	1.05	8.67	1.13	4.16	1.37	16.37	147
Male condom	0.00	0.00	1.77	0.00	2.03	0.00	5.59	9.38	51
Rhythm	0.61	1.13	0.89	1.39	4.26	0.00	0.00	8.27	150
LAM	0.00	0.00	0.00	0.00	24.93	0.00	1.76	26.68	47
Withdrawal	3.93	0.00	10.26	0.00	18.33	0.00	0.00	32.52	37
All methods	0.33	0.24	1.30	5.08	2.97	0.84	0.73	11.50	1215

**Table 3:** Contraceptive discontinuation rates at 6 months and associated reasons (*N*=574)

Discontinuation at 6 months postpartum									
Reasons for discontinuation									
Contraceptive method	Method failure	Desire to become pregnant	Other fertility related reasons	Side effects	Wanted more effective method	Other method related	Other/DK	All reasons	Unweighted N
IUD	0.00	0.00	0.00	8.34	0.00	0.00	0.00	8.34	73
Injectables	0.40	0.37	2.41	16.91	1.40	1.48	1.68	24.64	351
Implants	0.00	0.42	0.00	3.42	0.00	0.00	0.00	3.84	359
Pill	0.00	0.00	2.47	14.23	2.90	4.47	1.37	25.44	147
Male condom	0.00	0.00	1.77	0.87	5.31	0.00	12.43	20.37	51
Rhythm	2.28	1.13	1.23	1.39	11.77	0.00	0.34	18.14	150
LAM	0.00	0.00	4.80	1.80	41.56	0.00	1.76	49.91	47
Withdrawal	3.93	0.00	10.26	0.00	35.13	0.00	0.00	49.32	37
All methods	0.64	0.38	1.87	8.35	6.04	0.99	1.15	19.41	1215

**Table 4:** Contraceptive discontinuation rates at 12 months and associated reasons (*N*=574)

Discontinuation 12 months postpartum									
Reasons for discontinuation									
Contraceptive method	Method failure	Desire to become pregnant	Other fertility related reasons	Side effects	Wanted more effective method	Other method related	Other/DK	All reasons	Unweighted N
IUD	0.00	0.00	0.00	9.06	0.00	0.00	0.00	9.06	73
Injectables	1.66	0.88	3.37	21.09	3.77	4.53	2.86	38.15	351
Implants	0.00	0.42	0.00	4.69	0.00	0.00	0.00	5.11	359
Pill	2.53	0.00	2.47	18.48	6.06	4.47	2.47	36.48	147
Male condom	**0.00**	2.05	1.77	8.15	16.84	0.00	21.68	50.49	51
Rhythm	3.30	1.13	1.88	4.99	14.96	0.00	0.34	26.60	150
LAM	4.67	0.00	4.80	1.80	63.09	0.00	1.76	76.12	47
Withdrawal	3.93	0.00	10.26	0.00	54.70	0.00	2.85	71.74	37
All methods	1.81	0.59	2.26	11.26	9.58	1.97	1.94	29.40	1215

### Multivariable analysis for determinants of contraceptive discontinuation at 3, 6 and 12 months postpartum

The determinants for contraceptive discontinuation across time points are shown in Table 5 (focusing only on the first Family Planning use since the birth). In multivariable regression model, age and type of method discontinued were independently associated with postpartum contraceptive discontinuation. Women aged 40 to 44 years had significantly lower odds [(OR: 0.118, 95% CI: 0.016, 0.883)] of contraceptive discontinuation at 3 months postpartum compared to their counterparts aged 16 to 19 years of age. Furthermore, women who reported using implants had significantly lower odds of contraceptive discontinuation at 3 months, 6 months and 12 months postpartum (OR: 0.095; 95% CI: 0.051,0.176), (OR: 0.142; 95% CI: 0.073,0.276) and (OR: 0.218; 95% CI: 0.100,0.473) respectively, compared to those using injectables. Women who reported using pills had lower odds of contraceptive discontinuation at 3 months (OR: 0.527; 95% CI: 0.285, 0.975), 6 months (OR: 0.489; 95% CI: 0.246, 0.973) and 12 months (OR: 0.230; 95% CI: 0.068, 0.779) respectively compared to those who discontinued injectables. Other factors were not significantly associated with contraceptive discontinuation at 3, 6 and 12 months postpartum **(**Table 5).

**Table 5:** Multivariable analysis for factors associated with contraceptive discontinuation at 3, 6 and 12 months postpartum

	Discontinued		
	3 months OR (95% CI)	6 months OR (95% CI)	12 months OR (95% CI)
Characteristics			
Number of months since birth to FP use	1.026[0.967,1.088]	1.027[0.965,1.093]	1.001[0.927,1.080]
ANC visit (4-visits vs. < 4)	0.649 [0.408,1.031]*	0.733[0.453,1.188]	0.974[0.542,1.749]
Postnatal attendance7days (Yes)	0.825[0.468,1.455]	0.666[0.362,1.222]	0.679[0.310,1.488]
Age years (reference = 16–19)			
20–24	0.358[0.060,2.125]	0.773[0.185,3.236]	2.041[0.224,18.636]
25–29	0.379[0.062,2.301]	1.053[0.243,4.561]	3.854[0.418,35.509]
30–34	0.31[0.048,2.012]	0.663[0.140,3.132]	1.937[0.190,19.784]
35–39	0.239[0.036,1.593]	0.51[0.101,2.581]	1.961[0.185,20.790]
40–44	0.164[0.019,1.422]	0.356[0.049,2.587]	1.681[0.096,29.540]
Education level (reference = none)			
Primary	0.667[0.237,1.875]	0.728[0.239,2.214]	0.811[0.179,3.672]
Secondary	0.768[0.260,2.265]	0.689[0.214,2.221]	1.017[0.210,4.928]
Higher education	0.715[0.190,2.695]	0.505[0.117,2.172]	0.849[0.127,5.677]
Number of living children	0.813[0.628,1.053]	0.824[0.633,1.073]	0.766[0.546,1.075]
Desire to get pregnant	0.782[0.453,1.351]	0.590[0.329,1.058]	0.657[0.303,1.425]
Received FP counselling during their ANC or PNC	1.037[0.650,1.656]	0.936[0.565,1.551]	0.973[0.527,1.797]
Support from the husband/partner for contraceptive use	0.79[0.498,1.255]	0.771[0.478,1.246]	0.725[0.401,1.309]
Wealth index (reference = poorest)			
Poorer	1.012[0.441,2.320]	1.049[0.465,2.367]	1.765[0.710,4.390]
Medium	1.039[0.539,2.005]	0.891[0.455,1.743]	1.52[0.684,3.378]
Richer	1.043[0.597,1.820]	0.751[0.422,1.336]	0.591[0.288,1.213]
Richest	1.149[0.484,2.726]	1.281[0.535,3.062]	1.262[0.448,3.555]
Method discontinued (reference = injectables)			
Implants	0.092[0.049,0.172]***	0.136[0.069,0.269]***	0.212[0.097,0.464]***
Pills	0.538[0.291,0.995]**	0.499[0.254,0.980]**	0.230[0.069,0.767]**
Condom	0.603[0.225,1.617]	0.799[0.281,2.269]	0.62[0.142,2.712]
Rhythm	0.386[0.215,0.693]***	0.617[0.339,1.121]	1.127[0.581,2.186]
Other	0.595[0.220,1.606]	0.547[0.181,1.656]	1.03[0.241,4.397]
Cons	13.695[1.607,116.692]**	4.295[0.646,28.575]	0.398[0.020,7.732]

***p < 0.001; **p < 0.05

## Discussion

This study examined the determinants of postpartum contraceptive discontinuation at 3, 6 and 12 months postpartum. Overall, 11.5% of all episodes discontinued at 3 months, 19.4% discontinued at 6 months and 29.4% discontinued at 12 months. Women aged 40 to 44 years had significantly lower odds of contraceptive discontinuation at 3 months postpartum compared to their counterparts aged 16 to 19 years of age. Furthermore, women who reported using implants and pills had significantly lower odds of contraceptive discontinuation at 3 months, 6 months and 12 months compared to injectable users.

The average number of months since child birth to first family planning use was 3.8 months, this was similar to what was observed in rural Ghana [23] where the average time of first family planning use following child birth was 3.5 months and in contrast to what was observed in Nairobi slums where initiation of contraceptives following child birth occurred 7 months after delivery [11]. The fact that women initiate contraceptive use early in our setting may be encouraging, however the type of methods used must be borne in mind because short contraceptive methods have been associated with high rate of discontinuation compared to long term acting contraceptives [13]. Thus, short-acting contraceptive methods do not guarantee adequate birth spacing and the prevention of unwanted or mistimed pregnancies.

The most common methods discontinued after postpartum initiation in this study were LAM, pill and injectable. This is consistent with the previous reports from Tanzania Demographic and Health Survey and Health and Demographic Surveillance System (HDSS) in Magu district in Tanzania [13, 20]. Our finding was different from Malawian study, where women who reported long-acting methods and injectable use at 3 months post-delivery were more likely to continue compared to those using pills, condoms, traditional methods [2]. It also differs from the South Africa that reported high rate of implant continuation at 12 months at a rate of 86% [7]. The difference in findings could be explained by the differences in characteristics between the studied population especially the cultural barriers. Furthermore, the high contraceptives discontinuation rate in our population calls for a need to provide women with education on contraception, increase access to contraceptives as these will facilitate women to have informed choice and decision towards contraceptive use. However, there may be a possibility of some women experiencing forms of coercion to adopt a LARC the immediate postpartum period and choosing later to have the method removed.

The lower odds of postpartum discontinuation among women aged 40–44 years in our study is consistent with previous report in Kenya by [11] where higher odds of contraceptive discontinuation among adolescent women at 3 months, 6 months and 12 months compared with the adults counterparts. Previous studies in Tanzania and Nepal also reported the effect of age and type of contraceptive methods used on post-partum contraception discontinuation [13, 24]. The high contraceptive discontinuation among young women could be explained by their fertility desire to have more children. Unlike the short-term contraceptives, the lower discontinuation rate for long-acting methods could be explained by its difficulty to remove which requires health care professionals with cost implications [25, 26].

### Strengths and limitations of the study

The strength of this study is that, being a community-based study may reflect the representation of what is happening at the ground in the general population. We also used a rigorous data collection methods to enhance validity for observed findings. Despite its strength, some limitations which need to be taken into account while interpreting our finding. First, being a cross-sectional in nature, the study cannot establish a causal effect. Secondly, we did not collect information on sexual resumption, menstrual resumption, partners and service related factors which would further explain the methods discontinuation during the course of time.

## Conclusions

Lactational amenorrhea, male condoms, injectables and pills users had the highest rates of discontinuation compared with implants and IUD users. Women aged 40 to 44 years had lower odds of postpartum contraceptive discontinuation at 3 months while implants and pills had lower odds of contraceptive discontinuation at 3 months, 6 months and 12 months. Addressing barriers to prolonged contraceptive use amongst younger women and knowledge on attributes of contraceptive methods and their potential side-effects is warranted. In addition, the programs should assist women to timely switch to a method of their preference when they discontinue the method that fail, do not meet their expectations or cause side-effects.

## Data Availability

The datasets from this study are readily available from the corresponding author on reasonable request.
